# Decision of Character

**Published:** 1882-06

**Authors:** 


### DECISION OF CHARACTER.

Without it, no man or woman was ever worth a button, nor ever can be. Without it, a man becomes at once a good-natured nobody; the poverty stricken possessor of but one solitary principle, that of obliging every-body under the sun, merely for the asking. He is like the judge who uniformly decided according to the views of the closing speech. Having no mind of his own, such a man is a mere cypher in society, without weight of character, and utterly destitute of influence. Such an one can never command the respect or even the esteem of men around him. All that he can command, is a kind of patronizing pity. The man to be admired, respected, feared, and who will carry multitudes with him, whether right or wrong, is he who plants his foot upon a spot, and it remains there, in spite of storm, or tempest, or tornado: the very rage of an infuriated mob but gives new inspiration to his stability of purpose, and makes him see that he is so much the more of a man.

Then again, what a labor-saving machine is this "*decision of character*," this thin pressed lip, in all the departments of life; the infant of a year knows its meaning well: children see it with intuition. Servants, the dullest of the dull, the veriest flaxen waddle, a week only, from "Fader Land" learns it at a glance. Why! this *decision of character*, this firmness of purpose, pays itself in any walk down Broadway. The little *match girl* doesn't repeat *matches please?* the ragged crossing sweeper doesn't take the pains to run half across the street after you; he knows better. Your own child does not repeat its request, however anxious to have it granted, and wifey herself soon learns "it's no use knocking at the door any more," if the first tap does not gain admission.

Then again, what a happy deliverance it is from that state of betweenity, which is amongst the most wearing of all feelings. Why half the people don't know the luxury of having made up one's mind irrevocably. What an amazing saving of time it is, of words, of painful listening to distressing appeals. Why, it is a positive benefit to the persons refused, for it enables them to decide without an effort, that further importunity is useless. But my brother, see to it, that your decisions be always right, first; and to guarantee that, you must have a sound head and a good heart—then may it well be like a Medo-Persian law—unalterable. But "*be kindly firm.*"

---

### COMFORT FOR THE POOR.

How grandly benign the first penal sentence on man—"*In the sweat of thy face shalt thou eat bread*, UNTIL THOU RETURN TO THE GROUND"! How blessed the necessity of having to labor for one's daily food. It is steady, moderate work, which gives health, and years, and honor, and success. And yet, perhaps nine out of ten of us, are looking and laboring for the great consummation of being placed in a position which shall require no labor, the fabled *otium cum dignitate;* forgetting, that in the evasion of the penalty, greater judgments come. The sentence is, we must work *until we die.* That is its meaning and intent, and there is within it blessings in disguise; blessings in our very punishments! greater blessings than if we succeed in escaping them! How incomprehensible, how broadly reaching is the love of HIM, with whom we have to do!

It is within the observation of all of us, that those who retire from a long and active business life, soon fall into disease and die; and scarcely a day passes, in which the observant physician does not see, that the difference to many between health and disease, is the distance between the drawing-room and the wash-tub—the distance between the quill and the crow-bar—while the instructive fact with its terrible warning stares us in the face, that "*the average duration of the life of men after* 'RETIRING FROM BUSINESS' *is less than three years.*"

